# Soil Organic Carbon Attenuates the Influence of Plants on Root-Associated Bacterial Community

**DOI:** 10.3389/fmicb.2020.594890

**Published:** 2020-11-09

**Authors:** Yang Zhou, Qing Yao, Honghui Zhu

**Affiliations:** ^1^State Key Laboratory of Applied Microbiology Southern China, Guangdong Provincial Key Laboratory of Microbial Culture Collection and Application, Guangdong Microbial Culture Collection Center (GDMCC), Guangdong Open Laboratory of Applied Microbiology, Guangdong Institute of Microbiology, Guangdong Academy of Sciences, Guangzhou, China; ^2^College of Horticulture, South China Agricultural University, Guangdong Province Key Laboratory of Microbial Signals and Disease Control, Guangdong Engineering Research Center for Litchi, Guangdong Engineering Research Center for Grass Science, Guangzhou, China

**Keywords:** plant-derived C, soil-derived C, root-associated bacterial community, culture matrix, plant species

## Abstract

Plant-derived carbon (PDC) released by roots has a strong effect on root-associated bacterial community, which is critical for plant fitness in natural environments. However, the freshly exuded PDC can be diluted by the ancient soil-derived carbon (SDC) at a short distance from root apices. Thus, the rhizosphere C pools are normally dominated by SDC rather than PDC. Yet, how PDC and SDC interact to regulate root-associated bacterial community is largely unknown. In this study, a grass species and a legume species were planted in two contrasting matrixes, quartz sand and soil, to assess the role of PDC and SDC in regulating root-associated bacterial community, and to explore whether SDC affects the influence of PDC on bacterial community in soil. Our results indicated that the legume plant showed significantly positive priming effect on soil organic matter decomposition but the grass plant did not. PDC significantly shaped bacterial community in sand culture as indicated by PCR-DGGE and high-throughput sequencing of bacterial 16S rRNA gene. Intriguingly, we found that dissimilarity of bacterial communities associated with two plant species and the percentage of specific OTUs in quartz sand were significantly higher than those in soil. Moreover, several biomarkers enriched by plants in quartz sand turned to be general taxa in soil, which indicated that SDC attenuated the regulation of bacterial community by PDC. Taken together, these results suggest that SDC interacted with PDC and the root-associated microbial community, thus acted as soil buffering component of biological process contributing to soil resilience. The importance of PDC in structuring rhizosphere bacterial community needs to be reconsidered in the context of wider contribution of other C pool, such as SDC.

## Introduction

Plants can support relatively active microbial community in rhizosphere compared to that in the bulk soil (Paterson et al., 2007; Dennis et al., 2010; Haney et al., 2015; Schneijderberg et al., 2020). Root exudates, typically comprising sugars, amino acids, carboxylic acids and diverse secondary substrates, appear to be the largest and most active fraction of rhizodeposits to support root-associated microorganisms (Dennis et al., 2010; Hu et al., 2018). Root exudates, as carbon (C) and nitrogen (N) sources, can be assimilated by microorganisms rapidly within a few hours or days (Dakora and Phillips, 2002; Dennis et al., 2010). This biological process results in a dynamic microbial community and a fast soil nutrient cycling (Huo et al., 2017). In addition to primary metabolites, different kinds of structurally complex plant root secondary metabolites can also regulate soil microbial community, plant-soil feedbacks and their response to soil organic matter (SOM) (Hamer and Marschner, 2005; Hu et al., 2018; Okutani et al., 2020). For example, the addition of plant secondary metabolite catechol into soil altered the soil bacterial community and the response of microbial community to priming effect of SOM decomposition (Hamer and Marschner, 2005). Consequently, it is well acknowledged that plants shape root-associated microbial community mainly via the rhizodeposits (Tian et al., 2020).

According to the mineralization rates of sugars and amino/organic acids and the average root growth rate, Dennis et al. (2010) indicated that the concentration of root exudates surrounding the root would be halved at a distance of 0.4–7.2 mm behind the meristem zone, and would decline to 1% at a distance of 2–47 mm, which suggests that considerable volume of soil contains both fresh plant-derived C (PDC) and ancient soil-derived C (SDC). In this scenario, C sources for microorganisms in rhizosphere include two fractions, PDC newly derived from living roots and SDC from soil ancient organic matter. Both PDC and SDC are available or potentially available for microorganisms but different microbial taxa have their preferences, resulting in two microbial functional types that compete for PDC in the rhizosphere. Namely, the r-strategy microorganisms tend to utilize exclusively PDC, while the K-strategy microorganisms mainly live on SDC although they are able to utilize PDC (Fierer et al., 2007; Trivedi et al., 2013).

When exposed to root exudates and relatively recalcitrant SOM, rhizosphere copiotrophic microorganisms preferentially utilize the relatively simple PDC, which leads to the regulation of rhizosphere microbial community by plant host (Zhalnina et al., 2018). However, the microbial decomposition of SDC is ongoing whether roots are present or not, and only a limited volume of soil can be affected by rhizodeposits. Actually, except for the relatively recalcitrant humus substances, SDC contains as well various components with different physicochemical properties and hence different degree of degradability and turnover time. The liable organic fractions of SDC, e.g., carbohydrates, organic acids and fats can be easily consumed by many microbes (Kononova, 2013). Thus, SDC can affect bacterial biomass and community due to the higher C content and the wider range of degradability degree than PDC (Ding et al., 2015). Therefore, we inferred that the regulation of root-associated bacterial community in soil by plant hosts might be misestimated, because (1) a large proportion of rhizosphere bacteria can live on SDC (Kononova, 2013); (2) some oligotrophic bacterial taxa preferred SDC to PDC (Bird et al., 2011). Actually, the rhizosphere effect on microbial community is the results of PDC and its interaction with SDC rather than PDC alone. It is important to note that almost all studies claiming to demonstrate the driving force of root exudates in structuring rhizosphere microbial community normally fail to exclude the influence of SDC.

Plants can impact the turnover rate of native SOM through the interaction of roots and soil organisms, which is called rhizosphere priming effect (Huo et al., 2017). Rhizosphere priming effect may be positive, negative or neutral, depending on plant species and soil conditions (Kuzyakov, 2002; Huo et al., 2017), which reflects the difference in microbial biomass and community structure in response to PDC and SDC. Considering the co-metabolism of PDC and SDC by microbial community in rhizosphere, we hypothesized that the effects of PDC from different plant species in soil on root-associated microbial community might be attenuated by SDC. By using the legume and the grass planted in quartz sand or soil, we aimed (1) to reveal the difference in bacterial community composition shaped by PDC of legume and grass, and (2) to explore whether SDC affects the influence of PDC on root-associated bacterial community in soil.

## Materials and Methods

### Experimental Design and Sampling

Quartz sand and soil were used as matrixes to grow the grass species bahiagrass (*Paspalum notatum* Flüggé, PN) and the legume species stylo [*Stylosanthes guianensis* (Aubl.) Sw., SG]. The soil was collected from a subtropical orchard (E112•54′19″, N 22•40′20″), and the detailed information were described previously (Zhou et al., 2017). The soil from 0∼20 cm top layer was air-dried and sieved through a 2 mm pore-sized mesh for later use. In brief, the soil was moderately acidic (5.42) with the SOM content of 1.04%, and the total nitrogen, phosphorus and potassium was 0.87 mg g^–1^, 0.89 mg g^–1^, and 15.9 mg g^–1^, respectively (Zhou et al., 2017). The quartz sand was commercially obtained, sieved through a 2 mm pore-sized mesh, and soaked in 2 M sulfuric acid for 48 h to remove the adhering organic C. Then the acid-washed sand was rinsed with water until the pH of the leachate was neutral (pH 6.8). Finally, the sieved soil and rinsed sand were autoclaved and dried.

To ensure a similar volume, 1 kg soil or 1.45 kg quartz sand were filled in home-made acrylic rhizoboxes (15 cm wide, 30 cm deep and 3 cm thick). The flat rhizoboxes allowed a high root density in order to reinforce the effect of roots on bacterial community. Extracted soil bacterial supernatant of the original fresh soil was added as inoculum (25% vol/mass) to each rhizoboxes according to Fægri et al. (1977). The commercially obtained seeds of PN and SG were surface disinfected (10% H_2_O_2_ for 15 min) and germinated on moist paper soaked with sterilized water. Five pre-germinated seeds were sown in each rhizobox. A completely randomized factorial experimental design with culture matrix (soil and sand) and plant species (PN, SG and unplanted control CK as bulk soil) as two factors was established. The soil without plant was used to evaluate the sole effect of SDC, and the sand without plant was also included to test randomized effect. Each treatment comprised five replicates. All the rhizoboxes were placed in glasshouse with natural day/night photoperiod and temperature (22∼30•C) and watered every two days to keep the moisture of 20% by weighing method. To ensure plant normal growth in quartz sand rhizoboxes and to keep similar growth to plants in soil culture, 50 ml (the first 15 days) or 100 ml (the remaining cultivation time) Hoagland nutrient solution were irrigated every two days.

After three months, soil/sand in each rhizobox was collected. All roots in the collected soil/sand were removed, and the soil was sieved with a 2 mm pore-sized mesh. Homogenized soil/sand was divided into two subsamples. One subsample was air dried to measure soil/sand total organic C (TOC), and the other one was stored at −80•C for molecular analysis. To ensure the same batch of electrophoresis of all the samples (conducting electrophoresis of all samples in one gel), we randomly selected three samples from the five replicates to conduct PCR-Denaturing gradient gel electrophoresis (DGGE) under our laboratory condition. For comparison between DGGE and high-throughput sequencing, the same three samples for DGGE were used to perform the high-throughput sequencing.

### Measurement of Soil/Sand TOC

All the visible roots were carefully removed from the air-dried samples before the measurement of TOC. The content of TOC in soil and sand was measured by titration after wet oxidation with H_2_SO_4_ and K_2_Cr_2_O_7_ (Tiessen and Moir, 1993).

### Extraction of Sand/Soil DNA, PCR-DGGE and Quantification of Bacterial 16S rRNA Gene

The DNA was extracted from soil or sand samples (0.5 g) with the PowerSoil^®^ DNA Isolation kit (MoBio Laboratories Inc.) following the manufacturer’s protocol. To obtain sufficient amount of DNA and to diminish bias of DNA extraction for each sample, 3 subsamples were used for DNA extraction, then the obtained DNA was mixed together to generate a composite DNA sample used for PCR-DGGE, qPCR and high-throughput sequencing.

Nested PCR was performed with 27F/1492R and GC-341F/518R as the first and second primer set to amplify bacterial V3 region of 16S rRNA gene (Zhou et al., 2017). PCR amplification was conducted in triplicate for each sample. Then the PCR products were electrophoresed on a D-Code Universal Mutation Detection System (Bio-Rad Laboratories, Inc. United States) with the 8% polyacrylamide gel for 16 h at a constant voltage of 70 V and at 60•C in a 45%∼70% denaturant gradient. Then the gels were stained with SYBR Green I and visualized with Molecular Imager^®^ Gel Doc^TM^ XR+ (Bio-Rad Laboratories). DGGE band optical density profile was analyzed with Quantity One software to present bacterial community composition for further analysis.

Soil/sand bacterial abundance were quantified by using 16S rRNA gene copy numbers by qPCR (Zhou et al., 2017). The qPCR assays were conducted in triplicate on a CFX96 Optical Real-Time Detection System (Bio-Rad Laboratories, Inc. United States). The PCR reaction solution contained 10 μl 2 × PCR buffer (iQ^TM^ SYBR Green Supermix, Bio-Rad), 1 μl of each primer (primer set Eub338/Eub518, 10 μM), 1 μl of template DNA, and sterile deionized water added to 20 μl. The qPCR conditions were as follows: an initial denaturation at 95•C for 5 min; 40 cycles of denaturation at 95•C for 15 s, annealing at 56•C for 30 s. The plasmid DNA containing one copy of bacterial 16S rRNA gene V3 region was obtained by TOPO TA cloning kit (Invitrogen) as standard. Standard plasmid with ten-fold dilution series were used to gain a standard curve and amplification efficiency. Melting curve analysis and agarose electrophoresis analysis were performed to confirm the specificity of amplification products. Amplification efficiency was 98.4%, with linear fitness R^2^ > 0.99.

### High-Throughput Sequencing of Sand/Soil Bacterial 16S rRNA Gene Amplicons

High-throughput sequencing of the bacterial 16S rRNA gene V3-V4 region was conducted to evaluate bacterial community with a higher resolution than DGGE. The primer set 341F/806R was used, with each reverse primer fused with a unique 6-mer barcode for each sample (Zhou et al., 2017). Firstly, PCR reactions were carried out with Phusion^®^ High-Fidelity PCR Master Mix (New England Biolabs). Then the PCR products were electrophoresed to obtain bright main strip between 400–450 bp. Mixture PCR products with equidensity ratios for each sample were purified with Qiagen Gel Extraction Kit (Qiagen, Germany). Sequencing libraries were generated using TruSeq^®^ DNA PCR-Free Sample Preparation Kit (Illumina, United States) following manufacturer’s recommendation and index codes were added. The library quality was assessed on the Qubit@ 2.0 Fluorometer (Thermo Scientific) and Agilent Bioanalyzer 2100 system. At last, the library was sequenced on an Illumina HiSeq 2500 platform and 250 bp paired-end reads were generated. The raw sequencing data were submitted to NCBI Sequence Read Archive (SRA) with accession number PRJNA434434.

Raw sequence reads were de-multiplexed, paired-end reads merged, quality-filtered, processed and analyzed by QIIME (Caporaso et al., 2010). Then the sequences were compared against the reference Gold database using UCHIME algorithm to detect chimera sequences (Edgar et al., 2011). Clean tags with a similarity threshold of 97% were clustered as OTUs by Uparse software (Edgar, 2013). The representative sequence for each OTU was picked and used for taxonomy annotation against the RDP Classifier and Greengenes database. Samples were rarefied to 28123 sequences prior to downstream comparison analysis of samples to eliminate bias of sequence depth.

### Statistical Analysis

Analysis of variance (ANOVA) was conducted in R (version 3.1.0; R Development Core Team, 2017) to test significant difference of soil/sand TOC content, copies of bacterial 16S rRNA genes (log transformed data) and bacterial community alpha diversity (*P* < 0.05). Data normality and homoscedasticity were tested using Shapiro-Wilk (Shapiro and Wilk, 1965) and Levene’s test (Brown and Forsythe, 1974) with “stats” and “car” packages before ANOVA (Fox and Weisberg, 2011). Pairwise comparisons between groups were performed using Turkey HSD *post hoc* test (Tukey, 1949) with packages “agricolae” (de Mendiburu, 2017). The effect of matrix and plant on bacterial alpha diversity were analyzed using two-way ANOVA in R as described above.

Sand/soil bacterial community structure regulated by PDC and SDC were estimated by DGGE profiling and 16S rRNA gene V3-V4 high-throughput sequencing. Venn software online^1^ was used to identify shared and specific OTU and the proportion changes from sand culture to soil culture. In brief, the rarefied OTU table was used as raw data; OTUs with relative abundance <0.01% were replaced with 0, and then OTUs occurred one time within each group were treated as 0. The recovered OTU table were used as input data to plot Venn diagram.

Pair-wise Bray-Curtis similarity was calculated and hierarchical clustering of bacterial communities (UPGMA algorithm) were conducted in PAST software (version 3.11; Hammer et al., 2001). Two-way permutational multivariate analysis of variance (PERMANOVA) were conducted with *adonis* function of vegan package (Oksanen et al., 2013) in R software to reveal the influence of culture matrix and plant species on bacterial community. The pair-wise dissimilarities among samples were visualized by heatmap in R software (version 3.1.0; R Development Core Team, 2017) using package “pheatmap” (Kolde, 2015).

Significantly enriched OTUs by PDC with planted PN and SG under sand culture (plant-associated biomarker) were elicited using Turkey-Kramer analysis of variance followed by multiple comparisons with Bonferroni correction in the Statistical Analysis of Metagenomic Profiles (STAMP) software package (Parks and Beiko, 2010). The soil treatment was also performed as described above. We defined the underestimated rhizosphere bacterial OTUs according to the following criterions: (1) the OTU was significantly enriched in PN or SG in sand; (2) the OTU abundance in PN or SG was not significantly different from unplanted control in soil; (3) the OTU was top ranked with high effect size (>85%) calculated in STAMP. For each matrix, the OTUs with comparable abundance from unplanted control were defined as general taxa. Then the abundances of the underestimated rhizosphere bacterial OTUs were visualized by histograms generated with ggplot2 (Wickham, 2016) in the R environment (version 3.1.0; R Development Core Team, 2017).

## Results

### TOC Content in Quartz Sand and Soil as Affected by Plants

**FIGURE 1:**
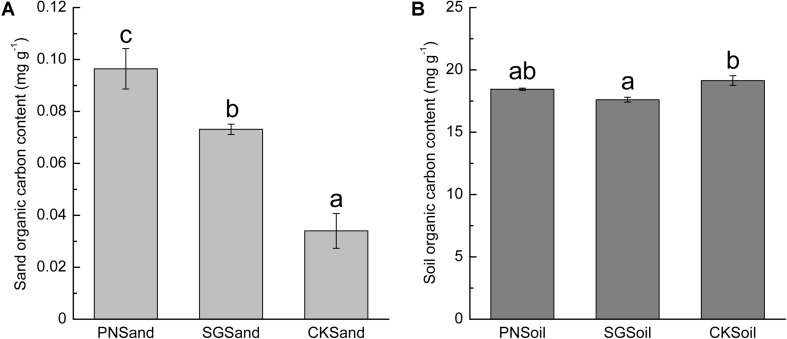
Sand organic C **(A)** and soil organic C content **(B)** as affected by plant. Different lowercase letters indicate significant difference (Tukey HSD *post hoc* test, *P* < 0.05) among three treatments of sand samples or soil samples. The error bars show calculated standard error of triplicate samples. PN, bahiagrass; SG, stylo; CK, the unplanted control.

Since the quartz sand was acid-rinsed before use and the visible root debris were carefully removed after sampling, the measured organic C content in sand samples of PN and SG can represent plant rhizodeposits, that is, PDC available for bacteria. Clearly, sand organic C in PN was significantly higher than that in SG (Figure 1A). In soil matrix, SG showed significantly positive priming effect on the decomposition of soil organic C, but PN only slightly decreased soil organic C content (Figure 1B). The sand organic C content was less than 0.4% of soil organic C (Figure 1).

### Bacterial Abundance and Community Diversity as Affected by Plants and Matrixes

**FIGURE 2:**
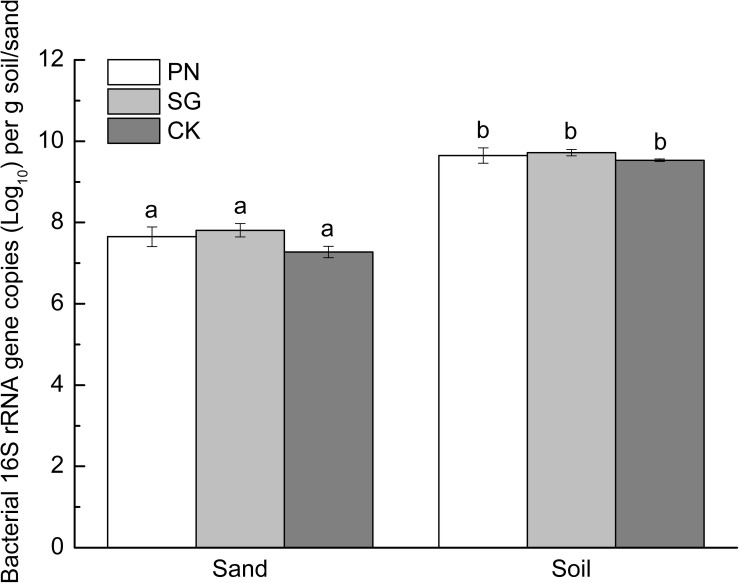
Abundance of sand/soil bacteria (revealed by 16S rRNA gene copy numbers) as affected by culture matrix and plant. Different lowercase letters indicate significant difference among the six groups (Tukey HSD *post-hoc* test, *P* < 0.05). The error bars show calculated standard error of triplicate samples. PN, bahiagrass; SG, stylo; CK, the unplanted control.

The soil matrix significantly increased bacterial 16S rRNA gene copy numbers compared to sand matrix, with the former averaged 4.29 × 10^9^ and the latter 3.78 × 10^7^ copies g^–1^. Moreover, within each culture matrix, plant did not show significant difference in bacterial abundance (Figure 2).

Both DGGE and high-throughput sequencing were performed to analyze the bacterial community associated with two plant species in two matrixes and their unplanted control. DGGE profiling indicated that both the estimated taxa numbers and Shannon diversity were significantly affected by matrix and plant (Table 1). The estimated taxa number and Shannon diversity in soil matrix were significantly higher than that in sand matrix, and plant growth significantly increased taxa number. Based on high-throughput sequencing data, we obtained a total of 583,559 high quality sequences (72% of the total 814,420) that could be classified for a median of 32,420 sequences per sample used in the subsequent analyses (range: 24,389∼43,889) with a mean read length of 412 bp. The high-quality reads were clustered into OTU, and then the low-abundance OTUs (<5 total counts) were discarded, resulting in 1,627 OTUs. Bacterial community diversity, as estimated by ACE (the estimated OTU numbers) and Shannon diversity, varied across culture matrixes with higher diversity values for soil culture (Table 1).

**TABLE 1:** Alpha diversity of soil bacterial community as revealed by DGGE and high-throughput sequencing.

	**DGGE**		**High-throughput sequencing**	
	**Taxa number**	**Shannon**	**ACE**	**Shannon**
PNSoil	34 ± 1 b	3.49 ± 0.04 cd	919 ± 21 b	7.19 ± 0.03 ab
SGSoil	36 ± 1 b	3.54 ± 0.01 d	903 ± 24 b	7.04 ± 0.05 ab
CKSoil	33 ± 1 b	3.46 ± 0.02 cd	968 ± 48 b	7.44 ± 0.04 b
PNSand	32 ± 0 a	3.42 ± 0.01 c	617 ± 35 a	6.44 ± 0.60 a
SGSand	28 ± 1 a	3.26 ± 0.03 b	694 ± 50 a	6.48 ± 0.27 a
CKSand	24 ± 1 a	3.12 ± 0.03 b	635 ± 52 a	6.34 ± 0.12 a
Matrix (M)	**<0.001**	**<0.001**	**< 0.001**	**0.004**
Plant (P)	**<0.001**	**<0.001**	>0.05	>0.05
Interaction (M × P)	**0.001**	**0.002**	>0.05	>0.05

*Each value represents the average ± standard error within group. Different lowercase letters indicate significant difference (Tukey HSD *post hoc* test, *P* < 0.05) among the six groups in each column. The *P* values indicated by Two-way ANOVA show the influence of matrix, plant and their interaction on alpha diversity, and the significant influence (*P* < 0.05) are highlighted by bold font. PN, bahiagrass; SG, stylo; CK, the unplanted control.*

**FIGURE 3:**
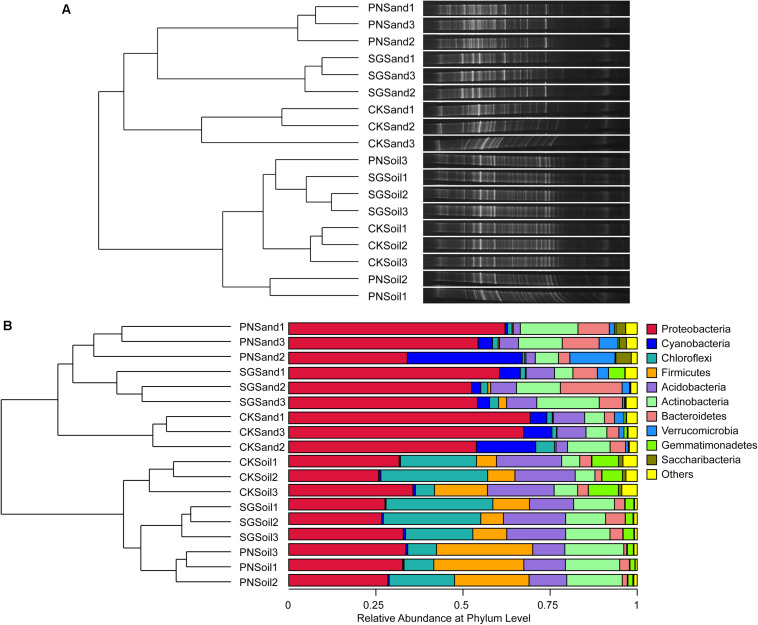
Soil bacterial community structure and hierarchical clustering based on community composition, as revealed by bacterial 16S rRNA gene V3 DGGE band optical density profiles **(A)** and 16S rRNA gene V3-V4 high-throughput sequencing OTU abundance profiles **(B)**. Hierarchical clustering dendrogram of bacterial communities were based on pairwise Bray-Curtis distance, and microbial community composition were visualized by DGGE bands and stacked bar charts of top ten phyla abundance from high-throughput sequencing. PN, bahiagrass; SG, stylo; CK, the unplanted control.

Two-way PERMANOVA test showed that culture matrix, plant presence, and their interactions significantly affected bacterial community (Table 2). The DGGE profiling revealed that sand and soil shaped distinct bacterial communities. Within sand samples, plant presence obviously changed bacterial community, but samples did not cluster well for soil samples, probably indicating that soil attenuated the shaping effect of plants on rhizosphere bacterial community (Figure 3A). With a high resolution, high-throughput sequencing data also showed the differences of bacterial communities, and samples clustered well according to culture matrix and plant species (Figure 3B). At phylum level, the relative abundance of Proteobacteria, Cyanobacteria and Verrucomicrobia were higher in sand samples, while the abundance of Acidobacteria, Firmicutes, and Chloroflexi increased in soil samples (Figure 3B).

**TABLE 2:** Percentage of variance in bacterial community composition (revealed by DGGE and high-throughput sequencing, respectively) explained by culture matrix and plant.

	**DGGE**			**High-throughput sequencing**		
	**Variance explained**	**F**	***P***	**Variance explained**	**F**	***P***
Matrix (M)	49.39%	62.90	<0.001	56.50%	47.08	<0.001
Plant (P)	16.96%	10.80	<0.001	15.80%	6.58	<0.001
Interaction (M × P)	24.23%	15.43	<0.001	13.20%	5.52	<0.001
Residual	9.42%	-	-	14.40%	-	-

*Data of band optical density profile from DGGE and OTU abundance profile from sequencing were analyzed by Two-way PERMANOVA in PAST (Bray-Cutis distance, permutation N: 9999).*

Selection of bacterial community in sand culture creates conditions in which microorganisms obtain substantial organic carbon from plants. While PN sand significantly enriched bacteria from *Novosphingobium*, *Verrucomicrobium*, *Asticcacaulis*, Rhizobiaceae and other taxa from Proteobacteria, Acidobacteria, TM6 and Saccaribacteria (OTU3, OTU36, OTU17, OTU107, OTU78, OTU215, OTU373, OTU693, OTU615, OTU720, OTU92, OTU222, OTU273, OTU49; Supplementary Table 1), SG sand mainly selected higher abundance of *Novosphingobium*, *Azospirillum* and other taxa from Alphaproteobacteria, Bacteroidetes (OTU3, OTU36, OTU72, OTU879, OTU1000, OTU89, OTU709, OTU1020, OTU899; Supplementary Table 1). While PN soil specially enriched OTUs from *Bacillus*, *Tumebacillus*, Alicyclobacillus, Rhodospirillales and some rare taxa (highlighted in pink and yellow background in Supplementary Table 2), SG soil mainly selected OTUs from Acidobacteria, *Inquilinus*, Saccharibacteria (highlighted in pink and green background in Supplementary Table 2).

### Changes in Bacterial Community From Sand Culture to Soil Culture

**FIGURE 4:**
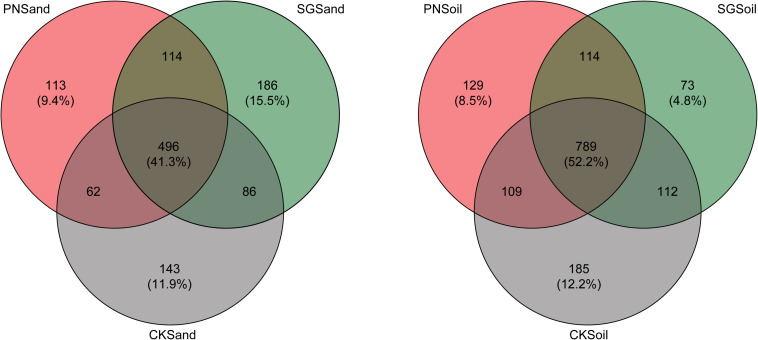
Venn diagram depicting numbers of shared and specific OTUs detected in sand and soil of PN, SG, and CK. Numbers in parentheses indicate the percentages of shared and specific OTUs. PN, bahiagrass; SG, stylo; CK, the unplanted control.

The specific and shared OTU numbers in each sample varied much depending on matrix and plant species. Generally, the shared OTU in sand were less than that in soil (41.3% vs. 52.2%), and the percentage of specific OTUs in sand (36.8%) was higher than that in soil (25.5%) (Figure 4). In detail, the percentage of PN-specific OTU in sand samples (9.4%) was higher than that in soil samples (8.5%), and the percentage of SG-specific OTU in sand samples (15.5%) was higher than that in soil samples (4.8%). Especially, the decreasing extent of specific OTU percentage for SG was much greater than that for PN when shifting from sand to soil (Figure 4).

**FIGURE 5:**
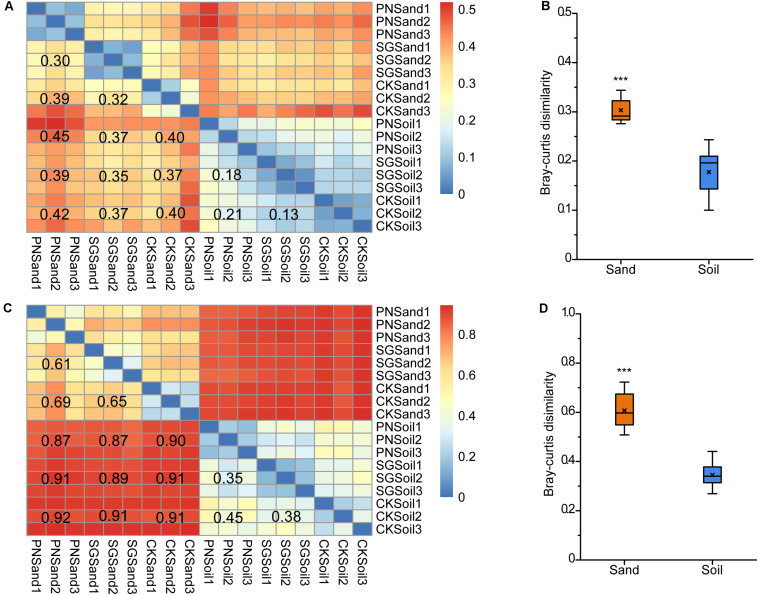
Bacterial community pair-wise dissimilarity as affected by culture matrix and plant. Heatmaps showing pair-wise Bray-Curtis dissimilarities revealed by 16S rRNA gene V3 DGGE band optical density profiles **(A)** and 16S rRNA gene V3-V4 high-throughput sequencing OTU abundance profiles **(C)**, and boxplots of community Bray-Curtis similarities between soil and sand from planted samples indicated by DGGE **(B)** and sequencing **(D)**. The values noted in the heatmap show the average dissimilarities of two groups. The “×” in the boxplots indicate the averaged dissimilarity values, and the “^∗∗∗^” mean significant difference between soil and sand planted samples followed by *t*-test (*P* < 0.05). PN, bahiagrass; SG, stylo; CK, the unplanted control.

We calculated the pair-wise Bray-Curtis dissimilarities to estimate the differences in bacterial communities in sand and soil. Consistent with the result of specific OTU percentage, the heatmaps showed that the community dissimilarity among sand samples were higher than that among soil samples, as revealed by both DGGE and sequencing analysis (Figures 5A,C). After the removal of unplanted samples, the dissimilarities between PN and SG in sand (0.30 for DGGE; 0.61 for sequencing) were significantly higher than those in soil (0.18 for DGGE; 0.35 for sequencing) (Figures 5B,D). Therefore, it appeared that soil matrix attenuated the shaping effect of plants on rhizosphere bacterial community by 40.0%∼42.6%.

**FIGURE 6:**
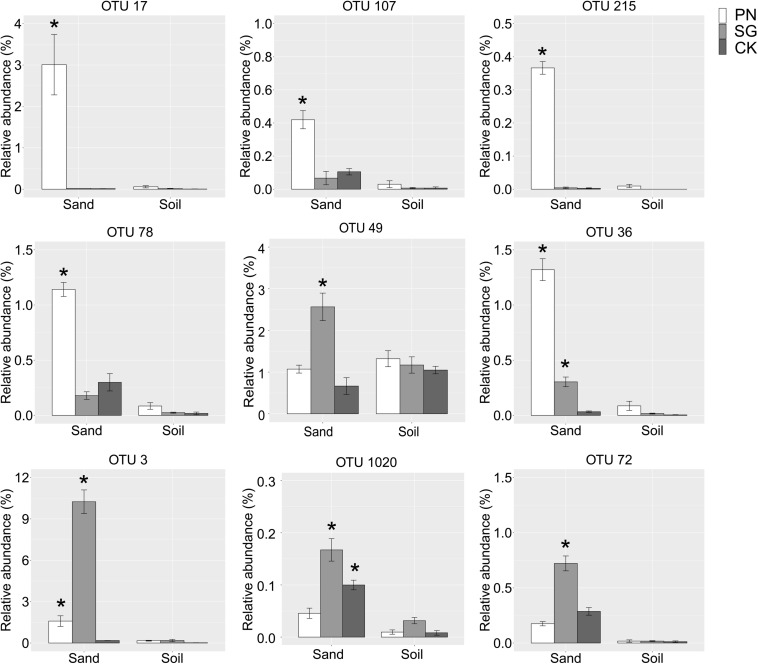
Relative abundance of the underestimated rhizosphere bacterial OTUs. These plant-associated OTUs were significantly enriched by plant in sand but turned to be general taxa with comparable abundance among groups in soil. The “*” means significantly enriched biomarkers by plant in sand compared to the unplanted control, as indicated by the STAMP software (corrected *P* value < 0.05, calculated using the Bonferroni correction). The error bars show calculated standard error of triplicate samples. PN, bahiagrass; SG, stylo; CK, the unplanted control.

We identified some underestimated rhizosphere bacterial OTUs for PN and SG. These OTUs were plant-associated biomarkers significantly enriched by plant in sand but turn to be general taxa with similar abundance among groups in soil. Specifically, the relative abundances of OTU 17 (Saccharibacteria), OTU 107 (Rhizobiales), OTU 215 (*Verrucomicrobium*), OTU 78 (*Bosea thiooxidans*), OTU 49 (*Phenylobacterium*) and OTU 36 (*Asticcacaulis*) for PN in sand were significantly higher than those of unplanted control, highlighting the six PN-associated biomarkers. In soil culture, however, the abundances of these OTUs drastically decreased and became general. Similarly, the underestimated rhizosphere bacteria for SG included OTU 3 (*Novosphingobium*), OTU 1020 (*Erythrobacteraceae*) and OTU 72 (*Defluviicoccus*) (Figure 6).

## Discussion

Previous studies have demonstrated that root exudates maintain and support a specific diversity of microorganisms in the rhizosphere of a particular plant species, indicating the role of PDC in shaping microbial community (Paterson et al., 2007; Zhalnina et al., 2018). On the other hand, SOM is one of the strong determinants of microbial community as well (Dennis et al., 2010; Delgado-Baquerizo et al., 2016). However, how these two forms of organic C and their interaction regulate soil microbial community remains unknown. Haichar et al. (2008) used stable isotope probing (SIP) to differentiate bacterial community assimilating root exudates from the bacterial community assimilating soil ancient organic C, which shed light on the difference in the two bacterial groups. In this study, we used sand and soil as culture matrixes to assess the role of PDC and SDC in regulating of bacterial community and to explore whether SDC affected the influence of PDC on bacterial community. Our results showed that PDC significantly affected bacterial community in sand with some taxa specifically enriched in grass or legume (Figure 3, Supplementary Table 1). Moreover, we found that SDC attenuated the strong regulation of root-associated bacterial community by PDC (Figures 3–5). The stronger shaping effect of plant on rhizosphere bacterial community than that of expected and the interactions with SDC reinforced the necessity of improving plant growth and health by rhizosphere holobiont engineering. For example, the plants and the associated microbes should be no longer viewed as individual but as a holobiont in future plant breeding program and crop growth management.

### Effect of PDC on Root-Associated Bacterial Community and Microbial Biomass

PDC can regulate root-associated bacterial community by providing nutrient and energy to enrich specific bacterial taxa (Chaparro et al., 2013; Yuan et al., 2016). PDC of grass and legume shaped different bacterial community and enriched specific taxa in sand culture in this study (Supplementary Table 1). Similarly, previous study reported that the difference in PDC composition, mainly in organic acid in the root exudates, explained a large proportion of the variations in bacterial communities associated with the legume lupin and the grass wheat (Weisskopf et al., 2008). In this study, unplanted treatment in sand culture (without PDC input) enriched some autotrophic bacteria, such as some OTUs from Cyanobacteria (OTU71, OTU62, OTU272, OTU262, OTU453, OTU548, and OTU597) and Chlorobi (OTU628), whose abundances were significantly higher than those in planted sand (Supplementary Table 1). Isolates in those phyla are usually known as autotrophic microorganisms (Garrity and Holt, 2001), which suggested that it was the PDC input that enriched the heterotrophic bacteria in planted sand. PN and SG in sand culture mainly enriched bacterial species known as plant growth promoting rhizobacteria (PGPR), which are capable of utilizing organic carbon, fixing nitrogen, being antagonistic against phytopathogens (Spaink et al., 1998; Kertesz and Kawasaki, 2010; Zhu et al., 2014; Shafi et al., 2017). We speculated that these plant-enriched bacteria detected in sand culture were directly nourished by PDC or benefitted from plant-microbe interactions independent on SDC, which can be confirmed by scrutinizing the plant response after the inoculating of each plant-enriched bacteria under axenic culture system in future study.

In addition to microbial community, the microbial biomass was also stimulated by liable C including PDC and other exogenous C (Blagodatskaya and Kuzyakov, 2008; Shi et al., 2011; Yuan et al., 2016). The fresh PDC can be assimilated by a wide range of microorganisms (Dennis et al., 2010), and the amount of released PDC may be important for the increase in microbial biomass (Groffman et al., 1996; Dornbush, 2007). In this study, bacterial 16S rRNA gene copies in planted samples were slightly higher than that in control (Figure 2), although the sand organic carbon content in planted samples was significantly higher than that in control (Figure 1). de Graaff et al. (2010) showed that high concentrations of exuded labile C (>3.6 mg C g^–1^) increased microbial gene copy numbers. However, the organic carbon recovered from planted sand matrix was less than 0.1 mg g^–1^ in this study (Figure 1), which might be too low to support abundant bacteria.

### SDC as Biological Buffering Component to Attenuate the Variation in Root-Associated Bacterial Community

SDC contributes significantly to soil resilience and soil buffering capacity (Ross et al., 2008; Gruba and Mulder, 2015). The direct buffering capacity of SDC can be reflected in chemical processes. For example, a large number of negative-charged moieties contained in SDC (e.g., carboxyl and phenolic hydroxyl) can bind the exchangeable cations, which contributes to soil acid buffering (Gruba and Mulder, 2015). Here, we demonstrated the attenuation of bacterial community dissimilarity among different plant species by SDC (Figures 3–5), providing evidence for the buffering capacity of SDC in biological processes. This buffering capacity in biological processes would result in a relatively stable microbial community and contribute to soil resilience in the conditions with PDC absent, e.g., reduced or no exudation input at night or in winter for deciduous plants. The biological buffering capacity mainly depends on the amount of SDC and its availability for most microorganisms (Dennis et al., 2010). Previous study reported that the effect of living roots on rhizosphere microbial community were moderate, and only less than 10% of microbial community responded to plant host (DeAngelis et al., 2008; Bird et al., 2011). SIP technique tracking the fate of PDC revealed that the bacterial population independent on PDC in the rhizosphere of rape was more than 6 times of that living on PDC (Haichar et al., 2008), which indicates that most rhizosphere bacteria live on SDC. However, the presence of SDC makes it complex to estimate the real role of PDC in structuring microbial community.

In this study, the amount of PDC from SG in sand culture was significantly lower than that from PN (Figure 1A). However, SG soil showed a stronger priming effect on SOM decomposition (Figure 1B). The shift from sand to soil sharply decreased the percentage of specific OTUs for SG, while the percentage of specific OTUs was slightly decreased for PN (Figure 4). These results may indicate that the soil bacterial community associated with legume plants is more susceptible to SDC. The quantity of the rhizodeposits may be one of the reasons. The roots of grass and legume respond in different ways to soil conditions (Watt and Evans, 2003; Weligama et al., 2008). The bulky root system of grass plants normally releases more C than that of legume plants in the same volume of culture matrix (Roumet et al., 2008; Ladygina and Hedlund, 2010). It is reasonable that the microbial community shaped by less amount of PDC (e.g., legume) might be more prone to be attenuated by SDC than that by more amount of PDC (e.g., grass). Moreover, PDC compositions of grass and legume are much different, and legume plants usually release exudates with lower C/N and containing more complex secondary metabolites. Therefore, it is reasonable to speculate that, compared to grass, the PDC composition of legume may be much different from the products of SOM decomposition. This can be another reason why more bacteria associated with legume are influenced during the shift from sand to soil.

We found some plant-associated biomarkers in sand matrix turned to be general taxa in soil matrix in this study (Figure 6), which indicates that some biomarkers nourished exclusively by PDC in sand become less competitive in soil, probably due to the dilution of PDC by SDC. The diffusion coefficient of root exudate in soil was low and most of root exudates, especially the organic acids such as citric and glutamic acids, were absorbed by the solid phase of the soil (Kuzyakov et al., 2003). In contrast, the inert media (e.g., quartz sand) do not have base exchange capacity and thus PDC in sand matrix can be easily obtained by microorganisms. However, it is difficult to link the specific taxa to their response to PDC and SDC in this study. Study on how specific bacteria respond to different components of PDC and SDC are needed in the future.

### The Interaction Between PDC and SDC to Change the Decomposition of SOM

The strong interactions make it difficult to separate the influences of SDC and PDC from each other in planted soil. Actually, what we usually observe regarding rhizosphere microbial community in soil system is the results of interactions between fresh PDC and ancient SDC. With SIP and the addition of synthetic root exudates, de Graaff et al. (2010) reported that the decomposition of SDC depended on the input concentration of root exudate. Previous studies reported that legumes, such as lupin and soybean, showed greater positive priming effect than the grass species wheat (Cheng et al., 2003; Wang et al., 2016). In this study, we also observed greater degree of decrease in soil TOC in stylo (legume) than that in bahiagrass (grass) (Figure 1B), which might reflect their different regulation of associated microbes and the interaction with SOM decomposition. One example is the regulation of N-fixing microorganisms by legume, namely, this symbiosis makes legume less depend on soil-derived N for growth and therefore compete less for N with microorganisms. These microbes become C-limited to a greater degree and are compelled to explore SDC, and therefore contribute to greater positive priming effect of SOM decomposition (Pausch et al., 2013).

## Conclusion

Both PDC and SDC regulated root-associated bacterial community. SDC promoted bacterial abundance due to the overwhelming amount in soil over PDC, however, the stimulation of bacterial abundance depended slightly on the input of fresh PDC in sand. PDC profile shaped the root-associated bacterial community structure, but the variation in bacterial community resulted from PDC among different plant species was attenuated by more than 40% by SDC in soil. When shift from sand to soil, legume plants sharply decreased more percentage of specific OTUs than grass plants did. This indicates a greater effect of legume PDC on bacterial community via the interaction with SDC, which resulted in a positive priming effect of SOM decomposition. Taken together, our results demonstrate that the shaping capacity of root-associated bacterial community by plants could be drastically underestimated only if plants are grown in soil. The rhizosphere effect needs to be reconsidered in the context of soil organic carbon in the future.

## Data Availability Statement

The raw sequencing data were submitted to NCBI Sequence Read Archive (SRA) with accession number PRJNA434434.

## Author Contributions

YZ performed the experiments. YZ and QY performed the data analysis. YZ, QY, and HZ wrote the manuscript. All authors conceived and designed the experiments, interpreted the results, and contributed to revisions and approved submission of the manuscript.

## Conflict of Interest

The authors declare that the research was conducted in the absence of any commercial or financial relationships that could be construed as a potential conflict of interest.

## Footnotes

1 http://bioinformatics.psb.ugent.be/webtools/Venn/
